# Mechanism of *CsGPA1* in regulating cold tolerance of cucumber

**DOI:** 10.1093/hr/uhac109

**Published:** 2022-05-17

**Authors:** Yan Yan, Sun Mintao, Ma Si, Feng Qian, Wang Yijia, Di Qinghua, Zhou Mengdi, He Chaoxing, Li Yansu, Gao Lihong, Yu Xianchang

**Affiliations:** 1 The Institute of Vegetables and Flowers, Chinese Academy of Agricultural Sciences, Haidian District, Zhongguancun South St, Beijing 100081, China; 2 Beijing Key Laboratory of Growth and Developmental Regulation for Protected Vegetable Crops, China Agricultural University, 2 Yuanmingyuan West Road, Haidian District, Beijing 100193, China

## Abstract

G proteins function directly in cold tolerance of plants. However, the framework of the Gα subunit in regulating cold tolerance remains to be explored. Here, we used protein interaction techniques to elucidate cold-related pathways regulated by CsGPA1. Suppression of *CsGPA1* decreased the cold tolerance of cucumber. Further protein interaction experiments showed that CsGPA1 interacted with Csa_4G663630.1 located in the cell membrane and nucleus and with CsCOR413PM2 located in the cell membrane. Csa_4G663630.1 was named CsCDL1 due to its 71% protein sequence similarity to AtCDL1, a positive brassinolide signal gene. Suppression of *CsGPA1* decreased the expression of most of brassinolide-related genes (including *CsCDL1*) under cold stress. Principal component and linear regression analyses showed that expressions of *CsGPA1* and brassinolide-related genes were positively correlated. Suppression of *CsCOR413PM2* also decreased cold tolerance of cucumber. The expression and protein content of *CsCOR413PM2* and *CsGPA1* in *CsGPA1*-RNAi and *CsCOR413PM2*-RNAi lines were determined under cold tolerance. Only *CsGPA1* silencing affected the expression and protein content of *CsCOR413PM2* during cold stress. Moreover, suppression of *CsGPA1* or *CsCOR413PM2* decreased Ca ^2+^ influx at low temperature and then decreased the expression of *CsICE*–*CsCBF*. These results indicated that the *CsGPA1*–*CsCOR413PM2*–Ca^2+^ axis regulated the expression of *CsICE*–*CsCBF* during cold stress. In conclusion, Our results provide the first framework of *CsGPA1* in regulating cold tolerance of cucumber, laying the foundation for further mechanistic studies of cold tolerance for Gα in cucumber.

## Introduction

The G protein heterotrimer, a protein complex consisting of Gα, Gβ, and Gγ subunits, is a core component of plant signal transduction [1]. The Gα subunit is involved in several signal transduction pathways, and in particular functions in morphogenesis and abiotic stress [2–4]. A breakthrough study found that the plasma membrane and endoplasmic reticulum local G protein regulator, CHILLING TOLERANCE DIVERGENCE1 (COLD1), conjugates rice G protein α subunit 1 (RGA1) to participate in the rice cold stress response by regulating the Ca^2+^ signal [5]. The COLD1–RGA1 complex mediates cold-induced intracellular Ca^2+^ influxes, leading to activation of cold-regulated (*COR*) genes [5].

Plant cells rely on special signaling pathways, such as calcium and brassinolide (BR), to enhance cold tolerance. Abiotic stresses, such as low and high temperature and salt stress, activate Ca^2+^ osmosis channels, leading to increased concentrations of free intracellular Ca^2+^, and these calcium signals transform external signals into various intracellular biochemical reactions, enhancing plant adaptation to abiotic stresses [6, 7]. Cold stress changes the fluidity of the plant cell membrane, while calcium signaling and other lipid membrane proteins can sense changes in cell membrane fluidity and activate the cold stress response, leading to enhanced expression of *ICE*, *CBF* and *COR* genes [8]. Therefore, calcium signaling is essential for the cold stress response of plants.

In addition to calcium, the BR signal is also important for cold tolerance in plants. In *Arabidopsis*, BR signaling enhances cold tolerance by activating the *ICE*–*CBF*–*COR* signaling axis [9]. Another study in *Arabidopsis* showed that, during the early stage of cold stress, BR signals alter the stability of ICE1 protein by controlling the activity of brassinosteroid-insensitive 2 (BIN2), thus balancing the cold tolerance of plants [10]. In tomato, inhibition of the BR signal could lead to increased reactive oxygen species, while spraying epibrassinolide or overexpression of *DWRF* improved the cold tolerance of tomato [11]. Moreover, BR signaling enhanced cold tolerance of tomato by activating *RBOH1* through the BZR1 transcription factor [12]. In cucumber, BR-mediated H_2_O_2_ signaling increased antioxidant enzyme activity to increase cold tolerance of seedlings [13–15]. Therefore, BR signals are widespread in the regulation of cold tolerance in plants.

In plants, the *COR* genes respond to low temperature [16]. *COR* is a type of gene that regulates cold tolerance. These genes encode osmotic and antifreeze proteins to protect plants from injury [17, 18]. Around 4000 *COR* genes have been identified in *Arabidopsis*, of which only a few hundred are regulated by C-repeat binding factors (CBFs). The promoters of these *COR* genes have *cis*-elements capable of recognizing CRT/DRE by CBF proteins, which contain conserved CCGAC sequences [19–21]. Other *COR* genes are regulated by CBF-independent proteins, such as HsfC1, ZAT12, and BZR1 [22–24]. *CBF*-independent pathways may therefore be required for regulating *COR* gene expression. In plants, most cold-regulated proteins are localized in the cytoplasm and nucleus. However, cold-regulated proteins located in the plasma membrane of cells are necessary for cold tolerance in plants [25]. Plasma membrane cold-regulated proteins include COR413 [26], COR47 [27], and WCOR410 [28]. COR413 includes five potential transmembrane domains (TMDs) [29]. The *COR413* gene encodes several transmembrane proteins, such as COR413PM in the plasma membrane of the cell and COR413TM in the locus chloroplast [30]. Recently, *cor413pm1* mutants of *Arabidopsis* was found to be less tolerant to low temperature than the wild type (WT) [31]. Overexpression of the *PsCOR413PM2* of *Phlox subulata* increased the expression of cold-stress-related genes *COR* and *CBF* in *Arabidopsis* [32]. Overexpression of *SikCOR413PM1* enhanced the cold and drought tolerance of transgenic tobacco [33]. In addition, overexpression of *LeCOR413PM2* improved the cold tolerance of tomato seedlings, and inhibiting *LeCOR413PM2* expression using RNA interference (RNAi) decreased the cold tolerance of seedlings [26]. Based on these studies, cold tolerance of plants is clearly related to COR413 protein in the plasma membrane.

Cucumber (*Cucumis sativus* L.) is an important cultivated horticultural crop in northern China, and its sensitivity to cold in winter has always been an urgent problem to be solved [34]. It is of theoretical and practical significance for cucumber breeding to improve its cold tolerance. Although the function of Gα in cold tolerance has been studied in *Arabidopsis* [35], rice [5], and tomato [36], the framework of *CsGPA1* in regulating cold tolerance of cucumber has not been reported.

Here, we explored low-temperature-related pathways regulated by *CsGPA1* by screening proteins that interacted with CsGPA1. We found that (i) CsGPA1 interacted with CsCDL1, a key BR signal transduction gene, and *CsGPA1* suppression weakens BR signaling in the regulation of cold tolerance; and (ii) CsGPA1 interacted with CsCOR413PM2 and suppression of *CsGPA1* decreased the expression and protein content of CsCOR413PM2; however, there were no differences in the expression and protein content of *CsGPA1* in *CsCOR413PM2*-RNAi lines compared with WT during cold stress. Furthermore, suppression of *CsGPA1* or *CsCOR413PM2* both decreased Ca^2+^ influx at low temperature and then decreased the expression of *CsICE*–*CsCBF*. Therefore, the *CsGPA1*–*CsCOR413PM2*–Ca^2+^ axis regulated the expression of *CsICE*–*CsCBF* during cold stress.

## Results

### Suppression of *CsGPA1* decreased cold tolerance of cucumber

**Figure 1:**
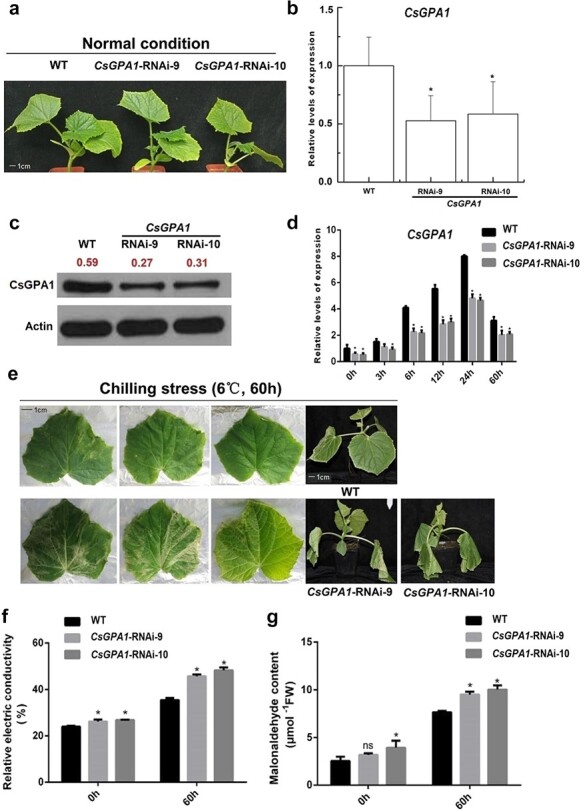
Functional verification of *CsGPA1* under cold stress. **a** Acquisition of *CsGPA1*-RNAi lines. Three treatments were set up: WT, *CsGPA1*-RNAi-9, and *CsGPA1*-RNAi-10, with five biological replicates for each treatment. **b**, **d** Expression of *CsGPA1* in WT and *CsGPA1*-RNAi lines. **c** Protein content of CsGPA1 in WT and *CsGPA1*-RNAi lines. Western blot of the WT and *CsGPA1*-RNAi lines using anti-CsGPA1 and anti-actin antibodies. The red numbers indicate the protein content of CsGPA1. **e** Functional verification of *CsGPA1*. **f** Relative electrical conductivity in WT and *CsGPA1*-RNAi lines. **g** Content of MDA in WT and *CsGPA1*-RNAi lines. All measured indices were set up with three biological replicates per treatment and time point. ^*^P < .05, significant difference between *CsGPA1*-RNAi lines and WT (Tukey HSD).

To investigate whether *CsGPA1* contributes to cold tolerance in cucumber, we obtained two independent transgenic RNAi lines to suppress *CsGPA1*: *CsGPA1*-RNAi-9 and *CsGPA1*-RNAi-10 (Fig. 1a). Real-time PCR (qPCR) and western blot (Fig. 1b, c) confirmed that the expression and protein content of *CsGPA1* were significantly decreased compared with WT in these RNAi lines. We then examined the response to cold stress in these knockdown lines by exposing WT and RNAi plants to 6°C for 60 hours. The results showed that while *CsGPA1* expression was significantly lower than that of WT at all timepoints, *CsGPA1* expression steadily increased over 24 hours, indicating that *CsGPA1* expression was indeed elevated in response to low temperature (Fig. 1d). The phenotypic effects associated with *CsGPA1* suppression were then examined in cucumber leaves by comparing the RNAi lines with WT, which revealed severe wilting under cold stress (6°C, 60 hours), whereas WT showed no obvious wilting (Fig. 1e). Determination of the relative electric conductivity (REC) and malondialdehyde (MDA) of cucumber seedlings (Fig. 1f and g) showed that REC was significantly higher in both RNAi lines compared with WT, as were the MDA values for RNAi-10, under ambient temperature (25°C). We then observed that both REC and MDA were significantly increased in the RNAi lines compared with WT following cold treatment. Together, these results suggested that *CsGPA1* contributed to cold tolerance in cucumber.

### Validation of *CsGPA1* interaction candidate proteins and its bioinformatic analysis

Since *CsGPA1* reached the highest expression levels in cucumber seedlings at 24 hours of cold treatment, leaves were collected at 24 hours and used to generate a cDNA library, which subsequently formed the basis for CsGPA1-cold-related protein interaction screens. Given previous studies showing that CsGPA1 was localized in the cell membrane [2], we performed split-ubiquitin yeast two hybrid (Y2H) assays to identify proteins that potentially interacted with CsGPA1. We identified 27 proteins interacting with CsGPA1. Among these 27 proteins, we speculated that Csa_4G663630.1 or CsCOR413PM2 might be involved in cold stress by bioinformatic analysis.

**Figure 2:**
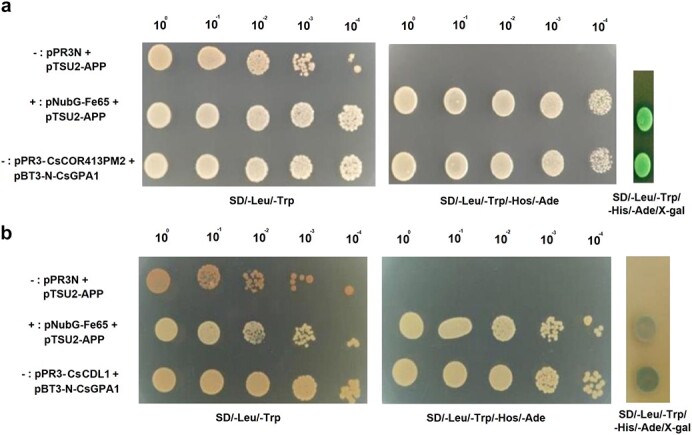
Split-ubiquitin Y2H validation of proteins interacting with CsGPA1. −, negative control; +, positive control; SD/−Leu/−Trp or SD/−Trp-Leu-His-Ade, dropout medium; 10^n^, dilution of yeast; X-gal, β-galactosidase.

**Figure 3:**
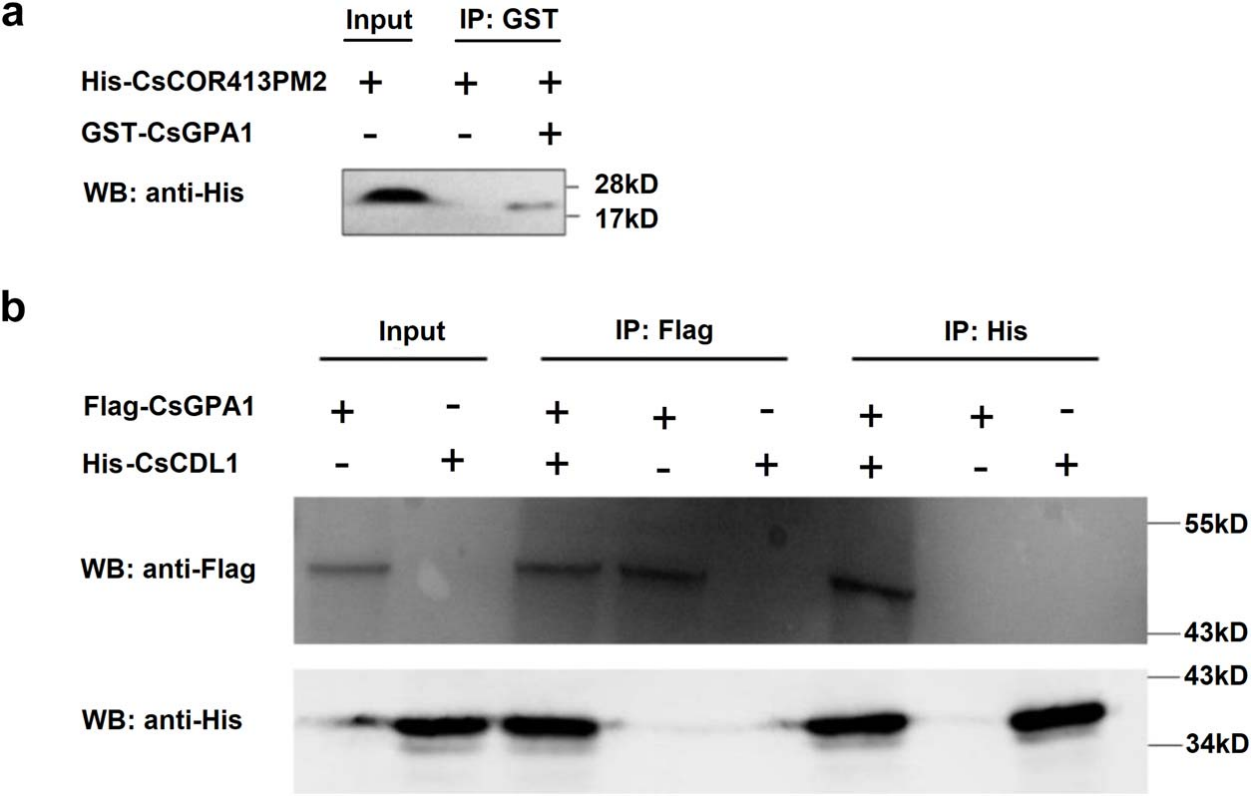
Pull-down validation of proteins interacting with CsGPA1. **a** Pull-down between His-CsCOR413PM2 and GST-CsGPA1. **b** Pull-down between His-CsCDL1 and Flag-CsGPA1 proteins.

Based on our screening of the CsGPA1 interaction library, we next verified the potential interactions between CsGPA1 with CsCOR413PM2 or CsCDL1 (Fig. 2). We first verified their interaction in split-ubiquitin yeast Y2H assays, and found that CsCOR413PM2 or CsCDL1 and CsGPA1 could grow on deficient media, indicating that CsGPA1 could interact with CsCOR413PM2 or CsCDL1 *in vivo* (Fig. 2a and b). Then we conducted a pull-down assay to determine whether they directly bind to one another *in vitro*. When tested with His antibody, only GST-CsGPA1/His-CsCOR413PM2 and Flag-CsGPA1/His-CsCDL1 combinations hybridized to produce bands, indicating that CsGPA1 interacted with CsCOR413PM2 or CsCDL1 (Fig. 3a and b).

Considering the interaction between CsGPA1 and Csa_4G663630.1 during response to cold stress and since Csa_4G663630.1 is annotated as a serine/threonine protein kinase, we next used Blastp to compare *Arabidopsis* non-redundant protein sequences to identify putative homologs. Csa_4G663630.1 shared 72% homology with *Arabidopsis* CDL1 (AT5G02800) in protein sequence (Supplementary Data Fig. S1), which has been shown to actively regulate BR signal transduction [37]. We therefore named Csa_4G663630.1 as CsCDL1.

To better understand the function of CsCOR413PM2, HMM software was used to search for proteins containing the WCOR413 (PF05562) domain in cucumber, tomato, rice, *Arabidopsis*, and *Nicotiana benthamiana*. Alignment of full-length sequences of the 15 COR413 proteins identified in these species were used to generate an unrooted neighbor-joining phylogeny (Supplementary Data Fig. S2). The three COR413 proteins in cucumber were separated into three different subfamilies in the tree. Cucumber has two COR413PM2 proteins, CsCOR413PM2 and CsCOR413PM2-2, but only CsCOR413PM2 (101203533) interacted with CsGPA1. Moreover, this protein belongs to the same subfamily and shares high amino acid sequence similarity with OsCOR413PM2 from rice, which has not been functionally characterized. This result indicated that *CsCOR413PM2* has not been previously described, nor has its function in the plant cold response been reported. In addition, we extracted the 1500 bp upstream of the 5′ UTR containing the *CsCOR413PM2* promoter region and found that it did not contain typical *cis*-elements of CRT/DRE, indicating that *CsCOR413PM2* may be a CBF-independent gene.

### Subcellular localization of CsCDL1 and CsCOR413PM2

**Figure 4:**
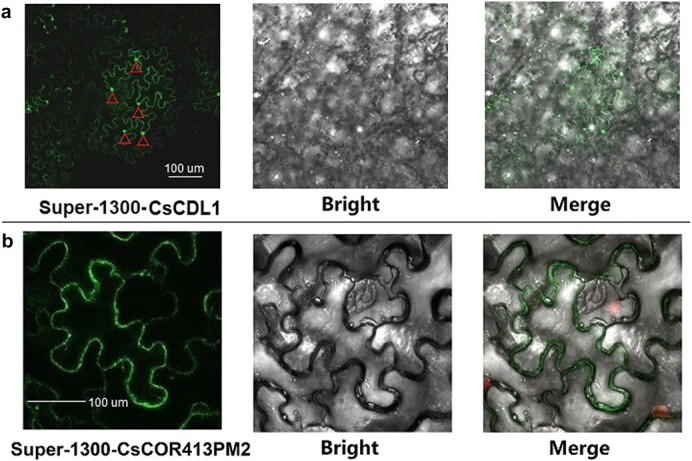
Subcellular localization of CsCDL1 and CsCOR413PM2. Subcellular localization of CsCDL1:GFP fusion protein (**a**) and CsCOR413PM2:GFP fusion (**b**). All fusions were expressed in *N. benthamiana* plants. Images at the far left show cells with GFP signal. In the middle are bright-field images of the same cells, and images at the end are overlays of the bright-field and fluorescence images. In (**a**) we labeled a total of five cells, and the five red triangles represent the nuclei of five cells. An irregular polygon surrounded by a green curve is the cell membrane (**a, b**).

Previous studies have shown that CsGPA1 is in the cell membrane [2], so we studied the localization of a CsCOR413PM2/CsCDL1-GFP fusion protein in cells by agroinfiltration into leaves of *N. benthamiana*. Observation by confocal microscopy showed fluorescence signal from CsCDL1:GFP expression at both cell margins and nucleus (Fig. 4a). The fluorescence signal from CsCOR413PM2:GFP expression in leaf epidermal cells could be found at the cell margins (Fig. 4b). These observations showed that CsCDL1:GFP was localized in the nucleus and cell membrane and that CsCOR413PM2:GFP was localized in the cell membrane.

### *CsGPA1* positively regulates the brassinolide signal to affect cold stress of cucumber

There is a potential interaction between CsGPA1 and CsCDL1 (Figs 2b and 3b), a BR signal transduction protein during the response to cold stress. Furthermore, several studies have found that the BR signal can enhance cold tolerance in cucumber [13–15, 38]. We thus hypothesized that CsGPA1 could potentially affect BR signaling in cucumber by interacting with CsCDL1 under cold stress.

**Figure 5:**
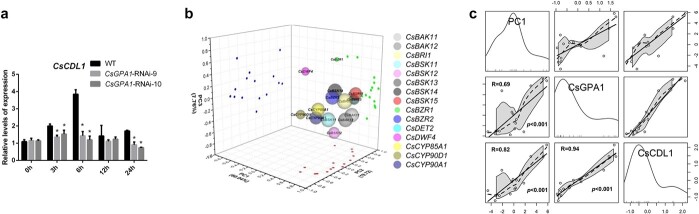
Relationship between *CsGPA1*, *CsCDL1*, and BR signal. **a** Expression of *CsCDL1* in WT and *CsGPA1*-RNAi lines. ^*^P < .05, significant difference between *CsGPA1*-RNAi lines and WT (Tukey HSD). **b** PCA of expression profiles for BR synthesis and signaling genes. The points on the different planes represent principal component (PC) values for PC1 (red), PC2 (green), and PC3 (blue). The size of the circle indicates the load of the gene on PC1**. c** Linear regression between PC1, *CsGPA1*, and *CsCDL1*. The gray shadow represents the 95% confidence interval of 2D data, and the diagonal graph represents the distribution of the corresponding data.

To test this possibility, we first detected *CsCDL1* expression under cold stress in both WT and *CsGPA1*-RNAi lines (Fig. 5a) and found that it reached maximum transcription in WT at 6 hours of cold treatment. *CsCDL1* expression almost decreased in the most time points over the course of cold treatment in plants with suppressed *CsGPA1*.

We then used qPCR to investigate BR synthesis and signaling genes in cucumber seedlings (Supplementary Data Fig. S3) and found that BR-related gene transcription was upregulated in both WT and RNAi lines, generally reaching maximum transcription levels at 6 hours of cold treatment, suggesting that BR signaling participated in the cold stress response. Compared with WT, only the *CsBZR1* and *CsBZR2* mRNA levels were significantly lower in RNAi lines at 0 hours of cold treatment, and the expression of other genes was not significantly different between WT and RNAi lines. Notably, with prolonged cold treatment, the expression of most BR-related genes decreased significantly compared with WT at 6, 12, and 24 hours, which suggested that *CsGPA1* suppression could result in decreased BR synthesis- and signaling-related gene expression during cold stress.

To further explore the relationship between *CsGPA1* and BR signaling, we next conducted principal component analysis (PCA) of BR-related gene expression (Fig. 5b) which revealed that PC1, PC2, and PC3 contributed 60.04, 19.72, and 7.76%, respectively, to the effects on BR gene expression. The component matrix showed that there were 11 genes with loads >70% in PC1 (Supplementary Data Table S2), indicating that PC1 replaced the expression of BR-related genes. Finally, a significant positive correlation was found between *CsCDL1* and PC1 (Fig. 5c), suggesting that *CsCDL1* was closely related to BR. Furthermore, a significant positive correlation was found between *CsGPA1* and *CsCDL1* and between *CsGPA1* and PC1, further indicating that *CsGPA1* may positively regulate the expression of BR synthesis and signaling genes at low temperature.

### Suppression of *CsCOR413PM2* decreased cold tolerance of cucumber

**Figure 6:**
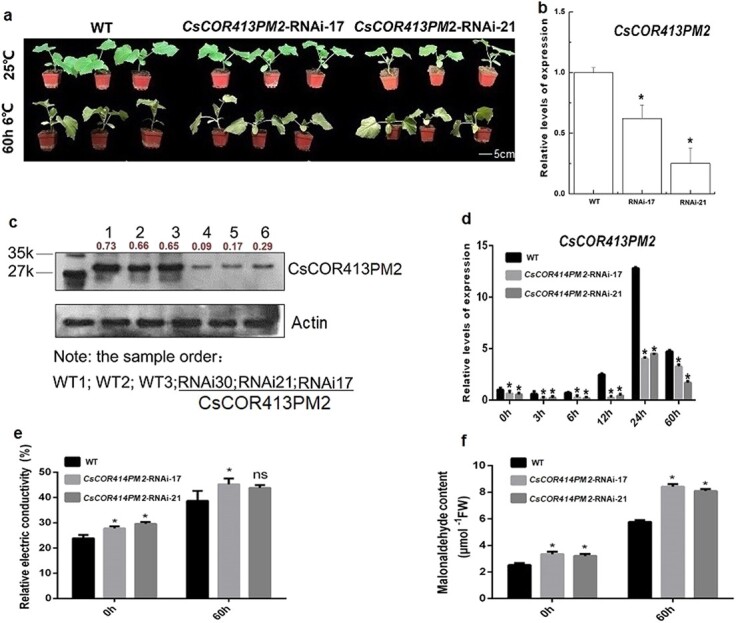
Functional verification of *CsCOR413PM2* under cold stress. **a** Acquisition of *CsCOR413PM2*-RNAi lines and functional verification of *CsCOR413PM2*. Three treatments were set up: WT, *CsCOR413PM2*-RNAi-17, and *CsCOR413PM2*-RNAi-21, with five biological replicates for each treatment. **b**, **d** Expression of *CsCOR413PM2* in WT and *CsCOR413PM2*-RNAi lines. **c** Protein content of CsCOR413PM2 in WT and *CsCOR413PM2*-RNAi lines. Western blot of WT and different transgenic lines for *CsCOR413PM2*-RNAi using anti-CsCOR413PM2 and anti-actin antibodies. The red numbers indicate the protein content. **e** REC in WT and *CsCOR413PM2*-RNAi lines. **f** MDA content in WT and *CsCOR413PM2*-RNAi lines. All measured indices were set up with three biological replicates per treatment and time point. ^*^P < .05 significant difference between *CsCOR413PM2*-RNAi lines and WT (Tukey HSD).

To verify whether *CsCOR413PM2* was involved in cold tolerance regulation of cucumber seedlings, we generated *CsCOR413PM2*-RNAi-17 and *CsCOR413PM2*-RNAi-21 transgenic knockdown lines (Fig. 6a). Analysis by qPCR (Fig. 6b) and western blot (Fig. 6c) confirmed that the expression and protein content of *CsCOR413PM2*, respectively, were both significantly decreased compared with WT in these RNAi lines. We next determined that the expression of *CsCOR413PM2* during cold stress was significantly lower in the RNAi lines compared with WT (Fig. 6d). Phenotypic analysis revealed that *CsCOR413PM2* silencing in cucumber leaves resulted in wilting under cold stress (6°C, 60 hours), while WT plants showed no obvious wilting or other symptoms of cold stress (Fig. 6a; Supplementary Data Fig. S4). Furthermore, both REC and MDA were detected to be significantly higher than WT in both RNAi lines under ambient temperature (Fig. 6e and f), while REC of RNAi-21 and MDA in both RNAi lines were significantly increased under cold stress compared with these values in WT (Fig. 6e and f). These results indicated that *CsCOR413PM2* was essential for cold tolerance in cucumber.

### Regulatory relationship between *CsGPA1* and *CsCOR413PM2*

**Figure 7:**
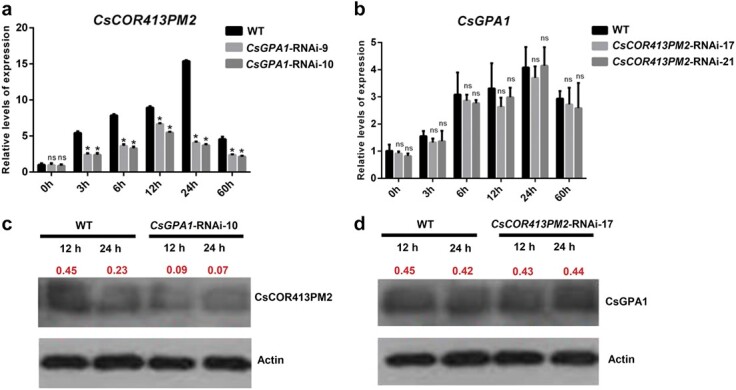
Relationship between *CsGPA1* and *CsCOR413PM2* in transcript and protein levels. **a** Expression of *CsCOR413PM2* in WT and *CsGPA1*-RNAi-10 during cold stress. **b** Expression of *CsGPA1* in WT and *CsCOR413PM2*-RNAi-17 during cold stress. **c** Protein content of CsCOR413PM2 in WT and *CsGPA1*-RNAi-10 during cold stress. **d** Protein content of CsGPA1 in WT and *CsCOR413PM2*-RNAi-17 during cold stress. Western blot of WT and different transgenic lines (*CsGPA1*-RNAi-10 and *CsCOR413PM2*-RNAi-17) using anti-CsCOR413PM2, anti-CsGPA1, and anti-actin antibodies. The red numbers indicate the protein content. All measured indices were set up with three biological replicates per treatment and time point. ^*^P < .05, significant difference between RNAi lines and WT (Tukey HSD).

To further identify the regulatory relationship between *CsGPA1* and *CsCOR413PM2* in transcript and protein levels, we determined the expression and protein content of *CsCOR413PM2* and *CsGPA1* in *CsGPA1*-RNAi and *CsCOR413PM2*-RNAi lines under cold stress (Fig. 7). We found that the expression and protein content of *CsCOR413PM2* significantly decreased in *CsGPA1*-RNAi lines compared with WT during the cold stress (Fig. 7a and c). However, there were no differences in the expression and protein content of *CsGPA1* in *CsCOR413PM2*-RNAi lines compared with WT during cold stress (Fig. 7b and d). The above results suggested that suppression of *CsGPA1* only affects the regulation *CsCOR413PM2* as part of the cold stress response.

### Suppression of *CsGPA1* or *CsCOR413PM2* decreased Ca^2+^ influx under cold stress, resulting in decreased expression of cold-related genes

**Figure 8:**
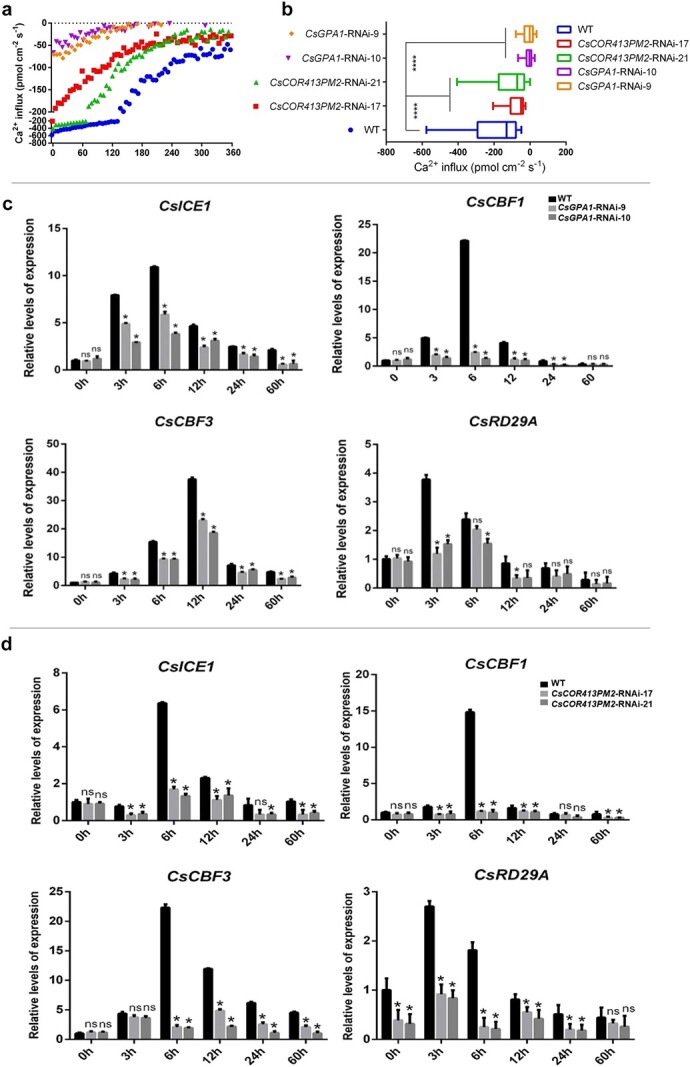
Ca^2+^ influx during low temperature and expression of cold-related genes in cucumber. **a** Ca^2+^ influx during low temperature for different treatments, including WT, *CsGPA1*-RNAi-9, *CsGPA1*-RNAi-10, *CsCOR413PM2*-RNAi-17, and *CsCOR413PM2*-RNAi-21. **b** Average Ca^2+^ influx for different treatments. **c**, **d** Expression of cold-related genes in WT, *CsGPA1*-RNAi, and *CsCOR413PM2*-RNAi plants. The negative sign (−) indicates Ca^2+^ influx. All measured indices were set up with three biological replicates per treatment and time point. ^*^P < .05, ^****^P < 0.001, significant difference between RNAi lines and WT (Tukey HSD).

Since cell membrane fluidity is affected by cold conditions, and calcium signals can sense this change and activate cold-stress-related genes in plants [8, 39], we next examined the relationship between *CsGPA1* or *CsCOR413PM2* and Ca^2+^ influx in cucumber roots using non-invasive microtest technology. We found that Ca^2+^ influx in RNAi lines was lower than that in WT under cold conditions and *CsGPA1*-RNAi lines showed the lowest Ca^2+^ influx (Fig. 8a). In addition, the average Ca^2+^ influx of RNAi lines was significantly different from that of WT under cold conditions (Fig. 8b). Moreover, *CsGPA1*-RNAi lines showed the lowest average Ca^2+^ influx under cold conditions (Fig. 8b).

Increased Ca^2+^ influx increases the expression of *ICE*–*CBF*–*COR* under cold stress [5, 39]. Based on these findings, we next detected the expression of genes induced by Ca^2+^ influx. Analysis by qPCR revealed that the expression of cold-related genes increased in both WT and RNAi lines in response to cold treatment, with *CsRD-29*, *CsICE1*, *CsCBF1*, and *CsCBF3* exhibiting maximum expression between 3 and 12 hours, while suppression of *CsGPA1* or *CsCOR413PM2* significantly decreased the expression of these four cold-related genes under cold stress (Fig. 8c and d).

## Discussion

Low temperature is a major environmental factor affecting plant geographical distribution [25]. To survive at low temperatures, plants have evolved a complex set of physiological and biochemical processes to adapt to low-temperature stress [18]. The G protein α subunit is involved in cold tolerance of *Arabidopsis* [35], tomato [36], and rice [5]. However, the framework of CsGPA1 in regulating cold tolerance of plants is still unclear. Here, cucumber, a cold-sensitive vegetable crop, was examined. We found that CsGPA1 interacted with CsCDL1, a key BR signal transduction gene, and suppression of *CsGPA1* weakened BR signaling in the regulation of cold tolerance; CsGPA1 interacted with CsCOR413PM2 and *CsGPA1* suppression decreased the expression and protein content of *CsCOR413PM2*. Furthermore, suppression of *CsGPA1* or *CsCOR413PM2* both decreased Ca^2+^ influx at low temperature and then decreased the expression of *CsICE*–*CsCBF*. Therefore, the *CsGPA1*–*CsCOR413PM2*–Ca^2+^ axis regulated the expression of *CsICE*–*CsCBF* during cold stress.

### *CsGPA1* affected cold tolerance of cucumber by regulating BR signal and *CsCOR413PM2*

G protein α subunit interacted with many proteins [4]. However, few studies have investigated the function and interactions of the Gα subunit with cold-related proteins, except for COLD1 in rice, which conjugates rice G protein α subunit 1 (RGA1) to participate in rice cold stress response by regulating calcium signaling [5]. The COLD1–RGA1 complex mediates cold-induced intracellular calcium influxes, leading to *COR* gene activation [5]. This study is the only report on the mechanism of Gα regulating cold stress in plants. Therefore, the mechanism of *CsGPA1* in regulating cold tolerance remains to be explored. In this study, suppression of *CsGPA1* significantly affected cold tolerance of cucumber, indicating that *CsGPA1* was involved in regulating cucumber cold tolerance (Fig. 1).

By screening the interaction proteins of CsGPA1, we explored the cold-stress-related pathways regulated by CsGPA1. CsGPA1 interacted with Csa_4G663630.1 (a serine lysine kinase) (Figs 2b and 3b) located in the cell membrane and nucleus (Fig. 4a). We further compared the protein sequence of *Arabidopsis* and found that Csa_4G663630.1 was 71% similar to AtCDL1, a positive BR signal gene, so it was named CsCDL1 (Supplementary Data Fig. S1). Further experiments showed that *CsGPA1* silencing leads to decreased *CsCDL1* expression (Fig. 4a). With the prolonged cold treatment, the expression of most BR-related genes decreased significantly compared with WT at 6, 12, and 24 hours, indicating that *CsGPA1* regulates BR synthesis and signal at low temperatures (Supplementary Data Fig. S2). Furthermore, the PCA and linear regression analysis showed a significant positive correlation between BR signal and the expression of *CsGPA1* (Fig. 5b and c). Taken together, these results suggest that *CsGPA1* can actively regulate the expression of BR synthesis and signaling genes at low temperatures. Our study is the first to show that the Gα subunit affected cold tolerance of plants by regulating BR signaling. Moreover, BR signaling is involved in regulating tolerance to cold stress [13–15, 38]. Therefore, we proposed that *CsGPA1* may mediate BR signaling under cold stress to regulate cold tolerance of cucumber. Additionally, studies have also shown that G protein α subunit affected U-box E3 ubiquitin ligase (TUD1) in *Arabidopsis*, and TUD1 mediated BR signaling transduction, regulated cell proliferation, and promoted plant growth and development [46, 47]. Previously, we have already found that, compared with WT seedlings, suppression of *CsGPA1* inhibits seedling growth and development and *CsGPA1* controls hypocotyl elongation and root growth by promoting meristem and cell size of hypocotyl and root tip cells in cucumber seedlings [2]. In this study, we also found that suppression of *CsGPA1* decreased expression of *CsBZR1* and *CsBZR2*, two BR-signaling-related genes under normal temperature, indicating that *CsGPA1* may affect growth and development of cucumber seedlings at normal temperature by influencing the BR signal. In general, the above results suggested that the G protein α subunit in plants mediated BR signaling to affect plant growth, development, and cold stress.

In addition to CsCDL1, we also found that CsGPA1 can interact with CsCOR413PM2 a cold-related protein (Figs 2a and 3a) located in the cell membrane (Fig. 4b). The Gα subunit participated in cold tolerance in *Arabidopsis* [35], tomato [36], and rice [5], and *COR413PM2* participated in cold tolerance in tomato [26]. However, the differences and the regulatory relationship between the two genes in plants have not been reported. In this study, we first verified the function of *CsCOR413PM2* in cucumber and found that suppression of *CsCOR413PM2* significantly decreased cucumber cold tolerance. We further compared phenotypes between *CsGPA1*-RNAi and *CsCOR413PM2*-RNAi lines after 60 hours of cold treatment (Figs 1e and 6a; Supplementary Data Fig. S4). We found that *CsGPA1* silencing led to more severe wilt and dehydration, suggesting that *CsGPA1* might be involved in multiple, functionally redundant cold-response pathways, such as mediating the BR signal, whereas the relatively low impact of *CsCOR413PM2* silencing suggests a comparatively minor role in cold tolerance. To further explore the regulatory relationship between CsGPA1 and CsCOR413PM2 in transcript and protein levels, we determined the expression and protein content of *CsCOR413PM2* and *CsGPA1* in *CsGPA1*-RNAi and *CsCOR413PM2*-RNAi, respectively, under cold stress (Fig. 7). We found that the expression and protein content of *CsCOR413PM2* significantly decreased in *CsGPA1*-RNAi plants compared with WT during cold stress (Fig. 7a and c). However, there were no differences in the expression and protein content of *CsGPA1* in *CsCOR413PM2*-RNAi lines compared with WT during the cold stress (Fig. 7b and d). The above results suggested that suppression of *CsGPA1* only affects the regulation of *CsCOR413PM2* as part of a cold-stress response.

In general, we first indicated that *CsGPA1* affected cold tolerance through regulating BR signal and *CsCOR413PM2* in plants.

### The *CsGPA1*–*CsCOR413PM2*–Ca^2+^ axis regulated the expression of *CsICE*–*CsCBF* during cold stress

The Gα subunit reportedly controls cold tolerance by mediating calcium signaling in rice [5] and studies also found that the calcium signal was essential for cold tolerance in cucumber [40, 41]. But it has remained unclear whether this pathway is conserved across other plant species, leading us to generate cucumber lines silenced for *CsGPA1* to examine its effects on cold tolerance (Fig. 7). We found that *CsGPA1* suppression significantly affected calcium influx at low temperatures (Fig. 8), suggesting that Gα-subunit-mediated calcium signaling in cold stress is conserved in both rice and cucumber. In addition, it is still not clear whether suppression of *COR413PM2* affects calcium signaling in plants now. In this study, suppression of *CsCOR413PM2* significantly deceased Ca^2+^ influx during cold stress (Fig. 8). Further comparison of Ca^2+^ influx between *CsGPA1*-RNAi and *CsCOR413PM2*-RNAi shows that *CsGPA1*-RNAi lines show lower Ca^2+^ influx than *CsCOR413PM2*-RNAi lines (Fig. 8), which may be a reason why suppression of *CsGPA1* resulted in weaker cold tolerance than suppression of *CsCOR413PM2*.

However, *CBF*-dependent or -independent genes can regulate *COR* expression [22, 23, 49, 50]. In this study, *CsCOR413PM2* was found to regulate *ICE–CBF* expression. *COR* genes have also been found to regulate expression of *CBF*. For example, one study found that repression of *COR27* and *COR28* by blue light negatively regulated *CBF* expression through crosstalk with *CCA1* function and with *PRR5* expression via unknown mechanisms [48]. In addition, *slyCOR413PM2* has been found to positively regulate *ICE–CBF* gene expression in tomato, but the mechanism is unknown [26]. However, in this study, suppressing *CsCOR413PM2* affected Ca^2+^ influx, and other studies also found that increased Ca^2+^ influx resulted in an increase in expression of *ICE*–*CBF*–*COR* under cold stress in plants [5, 45] (Fig. 8). Therefore, we next detected the expression of cold-stress-related genes that are induced by Ca^2+^ influx [5, 45]. *CsGPA1* or *CsCOR413PM2* knockdown decreased Ca^2+^ influx then decreased the transcriptional regulation of these four cold-related genes under cold stress (Fig. 8c and d). Therefore, *CsCOR413PM2* regulated *ICE*–*CBF–COR* expression through the Ca^2+^ signal. Since *CsGPA1* can control *CsCOR413PM2* transcription and translation, and both *CsGPA1* and *CsCOR413PM2* can regulate Ca^2+^ influx, the expression of *ICE*–*CBF* can be regulated by the *CsGPA1*–*CsCOR413PM2*–Ca^2+^ signal axis to affect cold tolerance of cucumber.

### Conclusions

We have provided the first framework of *CsGPA1* regulating cold tolerance of cucumber. We found that (i) CsGPA1 interacted with CsCDL1, a key BR signal transduction gene, and suppression of *CsGPA1* weakens BR signaling in the regulation of cold tolerance; and (ii) CsGPA1 interacted with CsCOR413PM2 and *CsGPA1* suppression could decrease the expression and protein content of *CsCOR413PM2*. Furthermore, suppression of *CsGPA1* or *CsCOR413PM2* both decreased Ca^2+^ influx at low temperature and then decreased the expression of *CsICE*–*CsCBF*. Therefore, the *CsGPA1*–*CsCOR413PM2*–Ca^2+^ signal axis regulated the expression of *CsICE*–*CsCBF* during cold stress.

## Materials and methods

### Plant material culture and treatment

WT and transgenic cucumber seeds were soaked in hot water at 55°C and constantly stirred until the temperature dropped to 30°C. Next, the seeds were soaked for 4–6 hours and then placed in a Petri dish with a diameter of 9 cm. Two layers of filter paper were added and moistened with 5 mL of bactericidal distilled water to keep them moist. When most of the seeds had germinated, they were sown with substrate (quartz sand:vermiculite = 1:1) in a plastic basin (15 cm $\times$15 cm). Cucumber seedlings were grown under 14 hours light (25°C)/10 hours dark (18°C) photoperiods (600 μmol m^−2^ s^−1^) with 70% relative humidity. When most of the seedlings had grown two leaves and one heart, seedlings at the same growth level were selected for cold stress. The WT and transgenic cucumber seedlings were treated at a low temperature of 6°C. Cucumber leaves were collected at low temperature for 0, 3, 6, 12, 24, and 60 hours, and then frozen in liquid nitrogen and finally stored in a refrigerator at −80°C. These samples were used to measure physiological indices or expression of genes (samples at each time point had three biological replicates).

### Real-time PCR

The methods of RNA extraction, cDNA synthesis, and qPCR were based on the previous study [2]. The qPCR primers are listed in Supplementary Data Table S1 (some of the qPCR primers were got from qPrimerDB). The gene expressions were calculated using the 2^-∆∆Ct^ method (reference gene: *CsActin*), and then R 4.02 software was used to analyze the significance of differences using Tukey’s HSD (*P* < .05). Prior to the PCA and regression analysis, the method of data standardization was based on our previously published paper [3]. PCA and regression analysis were performed on the gene expression data using R 4.02 software.

### Gene cloning and construction of RNAi vector

*CsGPA1* cloning and construction of *CsGPA1*-RNAi vector were according to our previously published paper [2]. The methods of *CsCOR413PM2* cloning and construction of *CsCOR413PM2*-RNAi vector were as follows. Forward and reverse fragments (200 bp) of *COR413PM2* were separately cloned, and then ligated to PHANNIBAL vector by using the NEBuilder^®^ HiFi DNA Assembly Cloning Kit (NEB #E2621S). The vector was then transformed into *Escherichia coli* DH5α competent cells, blue-white spot screening was performed, and the correct positive *E. coli* clone detected by PCR was sequenced. We obtained PHANNIBAL-COR413PM2-hairpin, which was cut with *SACI* and *PSTI* enzymes, and then we cloned the fragment into the same locus of pCAMBIA1300 vector to obtain pCAMBIA1300-COR413PM2-hairpin. The primers used in the study are shown in Supplementary Data Table S1.

### Subcellular localization

The full lengths of *CsCOR413PM2* and *Csa_4G663630.1* were amplified by using the specific primers shown in Supplementary Data Table S1. After digestion with HindIII and KpnI, the fragment was attached to the HindIII/KpnI site in Super-1300-GFP. Methods for subcellular localization were according to Huang *et al*. [42].

### Determination of malondialdehyde and relative electric conductivity

The MDA content was determined based on the method of Murshed *et al*. [43]. The REC was determined according to the method of Jiang and Zhang [44]. The calculation formula was as follows:

$$ \mathrm{REC}\left(\%\right)=\left({EC}_1/{EC}_2\right)\times 100\% $$

where EC_1_ and EC_2_ refer to the initial and final electrical conductivity, respectively. The data were analyzed using R 4.02 software for Tukey’s HSD (*P* < .05).

### Ca^2+^ influx detection

Calcium fluxes in roots were determined by using the non-invasive microtest (Younger LLC, Amherst, MA 01002, USA). Test solution at 0°C was used for the cold treatment. The data were analyzed using R 4.02 software for Tukey’s HSD (*P* < .05).

### Construction of a cold-related cDNA library of cucumber

The expression of *CsGPA1* reached its maximum after seedlings were treated with cold stress for 24 hours. Leaves at this time point were collected to construct a cDNA library that might be associated with *CsGPA1* in response to low temperature. The method of cDNA construction was based on our previous study [3].

### Split-ubiquitin yeast two-hybrid system

#### pBT3-N-CsGPA1 bait vector construction and yeast transformation

The method was based on our previous study [3].

#### Self-activation identification of decoy proteins

The plasmid combination pBT3-N-*CsGPA1*/pPR3-N was co-transformed into yeast NMY51. The mixture was coated on an SD−Trp−Leu medium and grown for 2–3 days. After positive clones were obtained, the monoclones were inoculated into SD−Trp−Leu medium for shake culture overnight. The bacterial fluid was placed on SD−Trp−Leu−His liquid medium containing 0, 5, 7.5, or 10 mmol L^−1^ 3-AT (3-amino-1,2,4-triazole), and then cultured at 30°C for 2 days.

#### Yeast two-hybrid assay and identification

The cDNA library plasmid of cucumber seedlings and bait pBT3-N-*CsGPA1* plasmid were extracted and co-transformed into NMY51. The transformation product was coated on SD−Trp−Leu−His medium + 10 mmol L^−1^ 3-AT. The plate was sealed with a membrane and cultured at 30°C for 3–4 days, and then colony growth was observed. Clones with β-galactosidase activity that could still grow normally on the SD−Trp−Leu−His−Ade + 10 mmol L^−1^ 3-AT deficient medium after three lines were plated were identified as positive clones. After plasmids were extracted from positive yeast colonies and transferred to *E. coli*, the plasmids were extracted from prey and then transformed into NMY51 yeast again with pBT3-N-*CsGPA1*. Y2H was carried out, with the SD−Trp−Leu−His−Ade + 10 mmol L^−1^ 3-AT deficient medium. Interacting genes were isolated from positive colonies growing on this medium. Additionally, pTSU2-APP and pNubG-Fe65 were co-transformed to NMY51 yeast as positive controls [45], while pTSU2-APP and pPR3-N were co-transformed to NMY51 yeast as negative controls [45].

### Pull-down assay

We designed primers according to gene sequence information on *CsGPA1*, *CsCOR413PM2*, and *Csa4G663630*. The RNA was extracted from leaves of cucumber seedlings, and cDNA was used as a template. After gel recovery, the target fragment was linked with pGEX4T1 and pET21a, and transformed into DH5α. The recombinant plasmids pET21a-*CsCOR413PM2/Csa4G663630.1* and pGEX4T1-*CsGPA1* were obtained by PCR identification and positive clone sequences. The recombinant plasmids pGEX4T1-*CsGPA1* and pET21a-*CsCOR413PM2/Csa4G663630.1* were separately transformed into strain BL21 (DE3) and we obtained the protein lysates. Finally, we used western blot to detect whether GST/FLAG/His-beads bind to His tag protein.

### Membrane protein extraction and western blot

The details of western blot and membrane protein extraction followed a previously published paper [2]. In addition, the N-terminal peptide of CsCOR413PM2 (7-LKMVTDSDAADLISSDC-22 + 16-ADLISSDLRELGNAARC-31) was synthesized by Beijing QiWei YiCheng Tech Co. Ltd. (Beijing, China) and used to produce a rabbit polyclonal antibody with CsCOR413PM2 (XP_004145347.1) as the antigen. ImageJ software (IJ152-WIN-Java8) was used to analyze the gray scale of western blot bands.

### Evolutionary tree construction

Proteins containing the WCOR413 (PF05562) domain in cucumber, tomato, rice, *Arabidopsis thaliana*, and tobacco were searched using HMM software, and then unrooted phylogenetic trees of the full-length COR proteins of different species were inferred using the neighbor-joining method in MEGA 7.0 software.

## Acknowledgements

This work was supported by the National Nature Science Foundation of China (32072650; 32102462), the National Key Research and Development Program of China (2018YFD1000800), and the Science and Technology Innovation Program of the Chinese Academy of Agricultural Sciences (CAAS-ASTIP-IVFCAAS), Ministry of Agriculture, China. We thank Shelley Robison, PhD, from Liwen Bianji, Edanz Editing China (www.liwenbianji.cn/ac) for editing the English of the manuscript.

## Author contributions

Y.Y., S.M.T., G.L.H., and Y.X.C. designed the study. Y.Y., F.Q., M.S., S.M.T., and D.Q.H. performed the experiments. Y.Y. collected the data. S.M.T. and Y.Y. performed all mapping and data analysis. S.M.T. and Y.Y. prepared and revised the manuscript. S.M.T., G.L.H., and Y.X.C. provided guidance on the whole study. All the authors approved the final manuscript.

## Data availability

The data are presented within the paper and supplementary files.

## Conflict of interest

There are no conflicts of interest to declare for authors.

## Supplementary data

Supplementary data is available at *Horticulture Research* online.

## Supplementary Material

Web_Material_uhac109

## References

[1] Gookin TE , Assmann SM. Significant reduction of BiFC non-specific assembly facilitates in planta assessment of heterotrimeric G-protein interactors. Plant J. 2014;80:553–67. 25187041 10.1111/tpj.12639 PMC4260091

[2] Yan Y , Zhang W, Li Y et al. Functions of CsGPA1 on the hypocotyl elongation and root growth of cucumbers. Sci Rep. 2018;8:15583. 30349017 10.1038/s41598-018-33782-4 PMC6197229

[3] Yan Y , Sun M, Li Y et al. The CsGPA1-CsAQPs module is essential for salt tolerance of cucumber seedlings. Plant Cell Rep. 2020;39:1301–16. 32648011 10.1007/s00299-020-02565-5

[4] Pandey S . Heterotrimeric G-protein signaling in plants: conserved and novel mechanisms. Annu Rev Plant Biol. 2019;70:213–38. 31035831 10.1146/annurev-arplant-050718-100231

[5] Ma Y , Dai X, Xu Y et al. COLD1 confers chilling tolerance in rice. Cell. 2015;160:1209–21. 25728666 10.1016/j.cell.2015.01.046

[6] Tunc-Ozdemir M , Tang C, Ishka MR et al. A cyclic nucleotide-gated channel (CNGC16) in pollen is critical for stress tolerance in pollen reproductive development. Plant Physiol. 2013;161:1010–20. 23370720 10.1104/pp.112.206888 PMC3560999

[7] Jiang Z , Zhou X, Tao M et al. Plant cell-surface GIPC sphingolipids sense salt to trigger Ca^2+^ influx. Nature. 2019;572:341–6. 31367039 10.1038/s41586-019-1449-z

[8] Zhu JK . Abiotic stress signaling and responses in plants. Cell. 2016;167:313–24. 27716505 10.1016/j.cell.2016.08.029 PMC5104190

[9] Eremina M , Unterholzner SJ, Rathnayake AI et al. Brassinosteroids participate in the control of basal and acquired freezing tolerance of plants. Proc Natl Acad Sci USA. 2016;113:E5982–91. 27655893 10.1073/pnas.1611477113 PMC5056081

[10] Ye K , Li H, Ding Y et al. BRASSINOSTEROID-INSENSITIVE2 negatively regulates the stability of transcription factor ICE1 in response to cold stress in *Arabidopsis*. Plant Cell. 2019;31:2682–96. 31409630 10.1105/tpc.19.00058 PMC6881119

[11] Xia XJ , Gao C-J, Song L-X et al. Role of H2O2 dynamics in brassinosteroid-induced stomatal closure and opening in *Solanum lycopersicum*. Plant Cell Environ. 2014;37:2036–50. 24428600 10.1111/pce.12275

[12] Fang P , Yan M, Chi C et al. Brassinosteroids act as a positive regulator of photoprotection in response to chilling stress. Plant Physiol. 2019;180:2061–76. 31189657 10.1104/pp.19.00088 PMC6670110

[13] Xia XJ , Wang Y-J, Zhou Y-H et al. Reactive oxygen species are involved in brassinosteroid-induced stress tolerance in cucumber. Plant Physiol. 2009;150:801–14. 19386805 10.1104/pp.109.138230 PMC2689980

[14] Cui JX , Zhou Y-H, Ding J-G et al. Role of nitric oxide in hydrogen peroxide-dependent induction of abiotic stress tolerance by brassinosteroids in cucumber. Plant Cell Environ. 2011;34:347–58. 21054437 10.1111/j.1365-3040.2010.02248.x

[15] Jiang YP , Huang LF, Cheng F et al. Brassinosteroids accelerate recovery of photosynthetic apparatus from cold stress by balancing the electron partitioning carboxylation and redox homeostasis in cucumber. Physiol Plant. 2013;148:133–45. 22998725 10.1111/j.1399-3054.2012.01696.x

[16] Guy CL , Niemi KJ, Brambl R. Altered gene expression during cold acclimation of spinach. Proc Natl Acad Sci USA. 1985;82:3673–7. 3858842 10.1073/pnas.82.11.3673 PMC397849

[17] Yamaguchi-Shinozaki K , Shinozaki K. A novel cis-acting element in an *Arabidopsis* gene is involved in responsiveness to drought low-temperature or high-salt stress. Plant Cell. 1994;6:251–64. 8148648 10.1105/tpc.6.2.251 PMC160431

[18] Shi Y , Ding Y, Yang S. Molecular regulation of CBF signaling in cold acclimation. Trends Plant Sci. 2018;23:623–37. 29735429 10.1016/j.tplants.2018.04.002

[19] Stockinger EJ , Gilmour SJ, Thomashow MF. *Arabidopsis thaliana* CBF1 encodes an AP2 domain-containing transcriptional activator that binds to the C-repeat/DRE a cis-acting DNA regulatory element that stimulates transcription in response to low temperature and water deficit. Proc Natl Acad Sci USA. 1997;94:1035–40. 9023378 10.1073/pnas.94.3.1035 PMC19635

[20] Gilmour SJ , Zarka DG, Stockinger EJ et al. Low temperature regulation of the *Arabidopsis* CBF family of AP2 transcriptional activators as an early step in cold-induced COR gene expression. Plant J. 1998;16:433–42. 9881163 10.1046/j.1365-313x.1998.00310.x

[21] Liu Q , Kasuga M, Sakuma Y et al. Two transcription factors DREB1 and DREB2 with an EREBP/AP2 DNA binding domain separate two cellular signal transduction pathways in drought- and low-temperature-responsive gene expression respectively in *Arabidopsis*. Plant Cell. 1998;10:1391–406. 9707537 10.1105/tpc.10.8.1391 PMC144379

[22] Vogel JT , Zarka DG, Van Buskirk HA et al. Roles of the CBF2 and ZAT12 transcription factors in configuring the low temperature transcriptome of *Arabidopsis*. Plant J. 2005;41:195–211. 15634197 10.1111/j.1365-313X.2004.02288.x

[23] Park S , Lee CM, Doherty CJ et al. Regulation of the *Arabidopsis* CBF regulon by a complex low-temperature regulatory network. Plant J. 2015;82:193–207. 25736223 10.1111/tpj.12796

[24] Li H , Ye K, Shi Y et al. BZR1 positively regulates freezing tolerance via CBF-dependent and CBF-independent pathways in *Arabidopsis*. Mol Plant. 2017;10:545–59. 28089951 10.1016/j.molp.2017.01.004

[25] Ding Y , Shi Y, Yang S. Advances and challenges in uncovering cold tolerance regulatory mechanisms in plants. New Phytol. 2019;222:1690–704. 30664232 10.1111/nph.15696

[26] Zhang L , Guo X, Zhang Z et al. Cold-regulated gene LeCOR413PM2 confers cold stress tolerance in tomato plants. Gene. 2021;764:145097. 32866589 10.1016/j.gene.2020.145097

[27] Lee SY , Boon NJ, Webb AA et al. Synergistic activation of RD29A via integration of salinity stress and abscisic acid in *Arabidopsis* thaliana. Plant Cell Physiol. 2016;57:2147–60. 27497445 10.1093/pcp/pcw132 PMC5434669

[28] Okawa K , Nakayama K, Kakizaki T et al. Identification and characterization of Cor413im proteins as novel components of the chloroplast inner envelope. Plant Cell Environ. 2008;31:1470–83. 18643950 10.1111/j.1365-3040.2008.01854.x

[29] Breton G , Danyluk J, Charron JBF et al. Expression profiling and bioinformatic analyses of a novel stress-regulated multispanning transmembrane protein family from cereals and *Arabidopsis*. Plant Physiol. 2003;132:64–74. 12746512 10.1104/pp.102.015255 PMC166952

[30] Lin C , Thomashow MF. DNA sequence analysis of a complementary DNA for cold-regulated *Arabidopsis* gene COR15 and characterization of the COR15 polypeptide. Plant Physiol. 1992;99:519–25. 16668917 10.1104/pp.99.2.519 PMC1080494

[31] Su C , Chen K, Ding Q et al. Proteomic analysis of the function of a novel cold-regulated multispanning transmembrane protein COR413-PM1 in *Arabidopsis*. Int J Mol Sci. 2018;19:2572. 10.3390/ijms19092572 PMC6165019 30158496

[32] Zhou A , Liu E, Li H et al. PsCOR413pm2, a plasma membrane-localized cold-regulated protein from *Phlox subulata*, confers low temperature tolerance in *Arabidopsis*. Int J Mol Sci. 2018;19:2579. 10.3390/ijms19092579 PMC6164191 30200233

[33] Guo X , Zhang L, Dong G et al. A novel cold-regulated protein isolated from *Saussurea involucrata* confers cold and drought tolerance in transgenic tobacco (*Nicotiana tabacum*). Plant Sci. 2019;289:110246. 31623784 10.1016/j.plantsci.2019.110246

[34] Yu JQ , Zhou YH, Huang LF *et al*. Chill-induced inhibition of photosynthesis: genotypic variation within *Cucumis sativus*. Plant Cell Physiol 2002;43:1182–8. 12407198 10.1093/pcp/pcf134

[35] Chakraborty N , Singh N, Kaur K et al. G-protein signaling components GCR1 and GPA1 mediate responses to multiple abiotic stresses in *Arabidopsis*. Front Plant Sci. 2015;6:1000. 26635828 10.3389/fpls.2015.01000 PMC4649046

[36] Guo X , Li J, Zhang L et al. Heterotrimeric G-protein α subunit (*LeGPA1*) confers cold stress tolerance to processing tomato plants (*Lycopersicon esculentum* Mill). BMC Plant Biol. 2020;20:1–16. 32847511 10.1186/s12870-020-02615-w PMC7448358

[37] Kim TW , Guan S, Burlingame AL et al. The CDG1 kinase mediates brassinosteroid signal transduction from BRI1 receptor kinase to BSU1 phosphatase and GSK3-like kinase BIN2. Mol Cell. 2011;43:561–71. 21855796 10.1016/j.molcel.2011.05.037 PMC3206214

[38] Wei L , Deng XG, Zhu T et al. Ethylene is involved in brassinosteroids induced alternative respiratory pathway in cucumber (*Cucumis sativus* L.) seedlings response to abiotic stress. Front Plant Sci. 2015;6:982. 26617622 10.3389/fpls.2015.00982 PMC4639706

[39] Liu Q , Ding Y, Shi Y et al. The calcium transporter ANNEXIN1 mediates cold-induced calcium signaling and freezing tolerance in plants. EMBO J. 2020;40:e104559. 33372703 10.15252/embj.2020104559 PMC7809786

[40] Liang W , Wang M, Ai X. The role of calcium in regulating photosynthesis and related physiological indexes of cucumber seedlings under low light intensity and suboptimal temperature stress. Sci Hortic. 2009;123:34–8.

[41] Zhang Z , Wuab P, Zhang W et al. Calcium is involved in exogenous NO-induced enhancement of photosynthesis in cucumber (*Cucumis sativus* L.) seedlings under low temperature. Sci Hortic. 2020;261:108953.

[42] Huang C , Hu G, Li F et al. Nbphan, a myb transcriptional factor, regulates leaf development and affects drought tolerance in *Nicotiana benthamiana*. Physiol Plant. 2013;149:297–309. 23387304 10.1111/ppl.12031

[43] Murshed R , Lopez-Lauri F, Sallanon H. Microplate quantification of enzymes of the plant ascorbate–glutathione cycle. Anal Biochem. 2008;383:320–2. 18682244 10.1016/j.ab.2008.07.020

[44] Jiang MY , Zhang JH. Effect of abscisic acid on active oxygen species antioxidative defence system and oxidative damage in leaves of maize seedlings. Plant Cell Physiol. 2001;42:1265–73. 11726712 10.1093/pcp/pce162

[45] Li Y , Jia Y, Bian Y et al. Autocrine motility factor promotes endometrial cancer progression by targeting gper-1. Cell Commun Signal. 2019;17:22. 30836961 10.1186/s12964-019-0336-4 PMC6402158

[46] Hu X , Qian Q, Xu T et al. The U-box E3 ubiquitin ligase TUD1 functions with a heterotrimeric Gα subunit to regulate brassinosteroid-mediated growth in rice. PLoS Genet. 2013;9:e1003391. 23526892 10.1371/journal.pgen.1003391 PMC3597501

[47] Wang L , Xu YY, Ma QB et al. Heterotrimeric G protein alpha subunit is involved in rice brassinosteroid response. Cell Res. 2006;16:916–22. 17117160 10.1038/sj.cr.7310111

[48] Li X , Ma D, Lu SX et al. Blue light and low temperature-regulated COR27 and COR28 play roles in the *Arabidopsis* circadian clock. Plant Cell. 2016;28:2755–69. 27837007 10.1105/tpc.16.00354 PMC5155342

[49] Thomashow MF . PLANT COLD ACCLIMATION: freezing tolerance genes and regulatory mechanisms. Annu Rev Plant Physiol Plant Mol Biol. 1999;50:571–99. 15012220 10.1146/annurev.arplant.50.1.571

[50] Zhao C , Zhang Z, Xie S et al. Mutational evidence for the critical role of CBF transcription factors in cold acclimation in *Arabidopsis*. Plant Physiol. 2016;171:2744–59. 27252305 10.1104/pp.16.00533 PMC4972280

